# High rates of suppurative otitis media among children attending urban clinics in Goroka, Eastern Highlands Province, Papua New Guinea: a cross-sectional study

**DOI:** 10.1016/j.lanwpc.2026.101807

**Published:** 2026-02-05

**Authors:** Celestine Aho, Tamara Veselinović, Rita Mark, Philip Teine, Tola Goina, Moses Laman, Rebecca L. Ford, Casparia Mond, William Pomat, Christopher G. Brennan-Jones, Michael J. Binks, Deborah Lehmann

**Affiliations:** a Infection and Immunity Unit, Papua New Guinea Institute of Medical Research, Goroka, Papua New Guinea; b Wesfarmers Centre for Vaccines and Infectious Diseases, The Kids Research Institute Australia, Perth, Australia; c Department of Audiology, Perth Children's Hospital, Perth, Australia; d UWA Centre for Child Health Research (affiliated with The Kids Research Institute Australia), The University of Western Australia, Perth, Australia; e Goroka Provincial Hospital, Goroka, Papua New Guinea; f School of Medicine and Pharmacology, The University of Western Australia, Perth, Australia; g School of Allied Health, Curtin University, Perth, Australia; h Child Health Division, Menzies School of Health Research, Charles Darwin University, Casuarina, Northern Territory, Australia; i Women and Kids Theme, South Australian Health and Medical Research Institute, Adelaide, South Australia, Australia

**Keywords:** Otitis media, Papua New Guinea, Infectious diseases, Child health

## Abstract

**Background:**

Otitis media (OM) is the leading cause of childhood hearing loss but its burden in low-middle-income countries like Papua New Guinea (PNG) is poorly understood. We aimed to determine the proportion of children aged ≤15 years attending clinics in Goroka, Eastern Highlands Province, PNG with OM and associated risk factors.

**Methods:**

In 2021–2022 any child attending outpatient clinics because they were sick or for ear screening or immunisation and accompanying siblings were eligible for enrolment into this cross-sectional study. Clinical and risk factor data were collected, ears examined by trained research nurses using otoscopy and tympanometry and findings independently reviewed by Australia-based audiologists. A child-level diagnosis was made based on the worst affected ear.

**Findings:**

Of 498 enrolled children, 68.1% attended for treatment, 15.3% for immunisation, 1.2% for ear screening and 15.5% were siblings. The proportion of children with any OM was similar among those attending because they were sick and other reasons (75.5% vs 71.4%) but suppurative OM (acute OM with/without perforation and chronic suppurative OM) was more common in children attending because they were sick (47.4% vs 16.9%). A tympanic membrane perforation was present in 22.1% of children. OM affected 79.0% of children <6 months; 45.4% of 1–2–year-olds had suppurative OM. Maternal smoking was associated with increased risk of any OM (adjusted odds ratio [aOR] 1.80, 95% CI: 1.08–3.00). Suppurative OM was associated with antibiotic use in preceding 30 days (aOR 1.78, 95% CI: 1.04–3.06).

**Interpretation:**

Children in the PNG highlands have among the world's highest burden of OM. Urgent public health action is required, including health worker training, regular ear screening, and strategies to prevent this under-reported disease in PNG.

**Funding:**

This project was funded by a Wesfarmers Centre for Vaccines and Infectious Diseases Deborah Lehmann Research Award.

**Research in context**

**Evidence before this study**

No comprehensive otitis media (OM) prevalence data from PNG have been published, despite reports of the highest rates of hearing loss globally being found in the Westen Pacific region, much of which may be attributable to OM. A study conducted in urban clinics in Port Moresby, the capital of Papua New Guinea (PNG), showed an OM prevalence of 18%. However, the study was hampered by lack of appropriate equipment (e.g. tympanometry) to satisfactorily diagnose OM. Prior to our study, no comprehensive research on OM prevalence had been conducted in PNG.

**Added value of this study**

This study has shown an alarmingly high burden of childhood middle ear disease in the Goroka area of the Eastern Highlands Province (EHP) of PNG. OM onset was early with ∼80% of children having OM and 10% a tympanic membrane perforation in the first 6 months of life. The proportion of children with any type of OM was highest in children under 3 years of age (86%) yet remained high (65%) in those aged 3–15 years. Overall, 38% of children had suppurative OM (acute OM with or without perforation or chronic suppurative OM (CSOM)). CSOM affected >10% of children, a rate far exceeding the World Health Organisation's 4% threshold for declaring an actionable public health emergency. Non-suppurative OM was prevalent in over half of the healthy children recruited into this study, suggesting it was largely asymptomatic.

**Implications of all the available evidence**

This study shows that children in the Goroka area of EHP in PNG have among the world's highest burden of OM. OM-related hearing loss detrimentally affects cognitive and language development, education, and social and emotional well-being. Enhanced screening and intervention capabilities are urgently required to address this unchecked public health crisis. Population-based studies are needed to determine the prevalence of ear disease in the wider PNG community, provide deeper insights into the underlying risk factors, and raise awareness of the importance of ear health in the region.

## Introduction

Otitis media (OM) is an infection of the middle ear and presents as a spectrum of diseases including otitis media with effusion (OME; glue ear), acute otitis media (AOM; bulging ear drum), and chronic suppurative otitis media (CSOM).^1^ In low-income settings children with OM often have no or mild symptoms and hence infection may go unnoticed.^2^ Without early detection, diagnosis and adequate management, OME may persist for months and AOM may cause perforation of the tympanic membrane (AOMwiP), which can lead to chronic suppurative otitis media (CSOM).^3^ Consequences of OM range from mild hearing impairment to severe disease associated with significant morbidity (e.g., mixed or sensorineural hearing loss or meningitis) and fatality from intracranial (e.g., meningitis or brain abscess) and extracranial complications (e.g., mastoiditis), especially in low-income settings.^3^^,^^4^

Hearing loss can affect a child's cognitive and language development, education, and social and emotional well-being in later life.^5^, ^6^, ^7^ According to the Global Burden Disease Study 2019, the Western Pacific has the largest group of people living with hearing loss.^8^ The WHO estimate that almost two-thirds of all the hearing loss in children younger than 5-years was attributed to OM.^8^

There are limited data on the prevalence of OM, related hearing loss and associated risk factors in Papua New Guinea (PNG). In a prospective study among children aged 6 months to 12 years attending urban clinics in the capital Port Moresby in 2014, clinical examination showed that 18% (70/395) had middle ear disease: 6% had AOM, 7% had CSOM but only 2 children were reported to have OME. Tympanometry was not available to assist in diagnosis.^9^ No risk factors were identified other than a history of recent antibiotic use among those attending with CSOM. In a neonatal 7-valent pneumococcal conjugate vaccine (7vPCV) trial conducted between 2005 and 2009 in the Asaro Valley, including Goroka town, in Eastern Highlands Province (EHP), 11% of children aged <18 months had visible middle ear discharge, suggesting a high burden of severe OM requiring immediate attention.^10^ Hearing was not assessed in either of these studies.

Globally, Indigenous children from Alaska, Greenland, Australia, New Zealand and Canada all suffer higher rates of OM than their non-Indigenous counterparts.^3^^,^^11^^,^^12^ Among Australian Aboriginal children an OM prevalence of 60–90% has been reported in rural and remote areas.^13^^,^^14^ In this population, the key risk factors for OM are young age, early and dense bacterial colonisation of the upper respiratory tract, upper respiratory tract infection (URTI), poor hygiene, crowded living conditions, limited access to clean water, smoking, and passive smoking.^6^^,^^15^ The three main aetiological pathogens of OM globally are *Streptococcus pneumoniae*, non-typeable *Haemophilus influenzae* (NTHi) and *Moraxella catarrhalis*.^16^, ^17^, ^18^, ^19^ Papua New Guinean children are exposed to *S. pneumoniae* and NTHi within days of birth and almost all infants are colonised with *S. pneumoniae* and *H. influenzae* by 3 months of age.^10^ During our clinical trial of neonatal 7vPCV, *S. pneumoniae* was isolated from 55% of 49 middle ear discharge swabs collected from children aged 3.5–18 months.^10^

Without baseline data on the burden of ear disease, government and non-government organisations, philanthropic foundations and global health partnerships lack the necessary guidance to develop strategies that will improve ear health in PNG and other lower-middle income countries. To guide effective OM prevention and treatment strategies in children living in urban and rural areas of the highlands of PNG, we conducted a cross-sectional study to evaluate the ear health of children aged ≤15 years attending primary health care clinics in Goroka town, EHP. The specific aims of this study were to determine the proportion of children with OM, severity of OM and associated risk factors among these children. We also investigated the microbiology of OM which will be reported separately.

## Methods

### Study design, location and population

Any child attending combined immunisation and acute care outpatient clinics (n = 3) in Goroka town were prospectively enrolled into a cross-sectional study between October 2021 and October 2022. Goroka is situated between 1500 and 1900 m above sea level. The 2021 population estimates for EHP, Goroka District and Goroka town were 784,535, 211,974 and 29,333 respectively.^20^ Outside the town people live in villages made up of small hamlets. Inhabitants are subsistence farmers with income from coffee and market sales. Housing infrastructure ranges from permanent houses, primarily in Goroka town, to semi-permanent or traditional housing made of bush materials in urban and rural settings. While higher levels of indoor air pollution have been documented in traditional bush material houses than in permanent and semi-permanent houses, high levels have been observed in all three house types, particularly during the wet season.^21^ Living standards vary widely both between and within urban, peri-urban and rural settings.

People travel 2–3 h to attend paediatric outpatient clinics in Goroka. Paediatric outpatient clinics in PNG are run by nurses who are trained to diagnose and treat common illnesses and provide immunisation based on national standard treatment guidelines.^22^ However, equipment such as otoscopes are generally unavailable. Children are referred from the town clinics to paediatricians or other specialists based some distance away at the Goroka Provincial Hospital as required. There is an Ear, Nose and Throat (ENT) clinic at the same hospital run by experienced specialist nurses (TG and late Inox Guhole). While there is an ENT specialist position at the hospital no ENT specialist was present during most of the study period. There are no qualified audiologists in PNG. Visiting Callan Services is currently training local staff in 5 centres including Goroka but were unable to visit during the COVID pandemic.

### Ethics statement

Ethical clearance for the study was granted by the PNG Institute of Medical Research (PNG IMR) Institutional Review Board (#2001) and the PNG Medical Research Advisory Committee (MRAC# 20.17). Written informed consent was obtained from parents or guardians before enrolment.

### Data collection

Any child aged ≤15 years presenting to one of the urban clinics for management of illness or immunisation or ear screening was invited to take part in the study and assessed against the inclusion and exclusion criteria by a PNG IMR study nurse. Thus, sick or healthy siblings were also eligible for inclusion in the study.

The inclusion criteria were age ≤15 years old presenting to one of the urban clinics for any reason and parental or guardian consent given. Children were excluded if suffering from a severe congenital abnormality (such as craniofacial malformations, congenital ear malformations or genetic syndromes) or if a parent/guardian declined or was unable or not present to give consent (i.e. child brought to the clinic by another relative or friend).

Following informed consent, demographic and risk factor data were collected and a clinical history taken by a research nurse. Parent-held child health books were generally available for research nurses to consult with regard to history of OM, antibiotic prescription and immunisation, although older children's earlier books that were full may have been left at home. Risk factors assessed included parents' education, housing, number of rooms in the house and number of people living in the house, washing facilities, immunisation history including 13-valent PCV (13vPCV, standard PNG schedule 1, 2 and 3 months of age),^22^ maternal smoking and recent antibiotic history (in the past 30 days). Each child had a general clinical examination followed by a detailed ear examination, which included i) otoscopy using a HearScope video-otoscope (HearX Group, Pretoria, South Africa) for visual inspection of the ear canal and tympanic membrane and ii) tympanometry to assess middle ear function using a Titan Middle Ear Analyser (1000 Hz or 226 Hz probe tone). The clinical examination including ear examination was performed by a research nurse and a preliminary diagnosis was made based on the data obtained and in accordance with the OM Guidelines for Australian Aboriginal and Torres Strait islander children.^23^ All data were entered onto a REDCap database. Following data entry, the clinical history, tympanometry, and video-otoscopy data were independently reviewed by audiologists in Perth, Western Australia, who assigned the final clinical diagnosis for each ear. Prior to study commencement, the Chief Investigator (CA) visited Australia and shadowed audiologists and ENTs and was trained to use the study equipment. This training was then brought back to PNG to train the research nurses, and additional online meetings were held with Australian audiologists, specifically pertaining the use of the study equipment and additional clinical training. Clinical ear health training was also provided by experienced specialised ENT nurses from Goroka Provincial Hospital (TG and Inox Guhole (deceased)).

During the study, regular online meetings between the PNG IMR team and audiologists and epidemiologist in Perth were used to provide clarification to finalise the ear health diagnoses. Final clinical diagnoses were coded according to the International Classification of Diseases version 10.^24^ Nasopharyngeal swabs from all children and swabs of ear discharge, if present, were collected for microbiological investigations which will be reported separately. Treatment and immunisations were provided according to standard schedules in PNG and children referred to a paediatrician or the ENT clinic as required.^22^ Amoxycillin was prescribed for 5 days for acute OM; if AOMwiP, ear cleaning with tissue spears was also recommended and demonstrated to the parent/guardian. Where acute discharge was deemed to have been present for >1 week, cotrimoxazole was prescribed. Children with symptoms or signs suggestive of mastoiditis were referred to the hospital immediately. For CSOM (ear discharge >2 weeks, based on history from parent or guardian or parent-held health book), ear cleaning with tissue spears was recommended and demonstrated to the parent/guardian and topical boric acid was prescribed and application was demonstrated by the research nurse.

### Final OM diagnosis

Visual inspection of the ear canal and tympanic membrane (TM) was recorded as still images or video recordings using a HearScope video-otoscope. The TM was classified according to visibility, position and presence or absence of a perforation.

Tympanometry was classified according to ear canal volume (ml), static compliance (ml) and peak tympanic pressure (daPa). 1000 Hz tympanograms were used for children <6 months of age, which was classified based on the presence of a peak (normal middle ear function, classified as a type A) or no peak (indicative of OM, classified as type B).^25^ Supplementary Table S1 outlines the classification used for 226 Hz tympanograms for children ≥6 months of age. Type A tympanograms were indicative of normal middle ear function, type B tympanograms indicative of OM, and type C indicative of eustachian tube dysfunction. A high-volume type B tympanogram was indicative of a perforation of the TM. If middle ear discharge was present, tympanometry was not performed but was classified as a high-volume B (i.e. perforation present). If the tympanogram could not be classified based on the trace obtained, tympanometry results were classified as unknown.

Based on otoscopy and tympanometry results as well as clinical history (e.g., duration of illness and fever), each ear was assigned a final diagnosis by the independent audiology team in Australia based on the OM Guidelines for Aboriginal and Torres Strait islander children.^23^ Ears were hierarchically classified as follows: normal, OME, AOM without perforation (AOMwoP), AOMwiP, dry perforation, CSOM or unknown/unclear diagnosis.^23^ Ears classified as having eustachian tube dysfunction by the research nurses were categorised as normal if associated with a type C tympanogram and as OME if associated with a type B tympanogram. Where an ear diagnosis was not possible or unclear, it was recorded as unknown. All children with a valid examination in at least one ear were included in the analysis. Where only one ear had a valid diagnosis, it was considered the worst ear. When reporting on proportion of children with bilateral OM, only children with a diagnosis in both ears were included.

### Sample size

Based on the recent ear health studies in Aboriginal children in Western Australia and the Northern Territory of Australia and the prevalence of ear discharge reported by Aho et al. in the Asaro Valley, we estimated that 40–50% of children would have OM (as determined by a type B tympanogram indicating fluid in the middle ear).^10^^,^^13^^,^^14^ To ensure statistically reliable estimates of the proportion of children with OM that would be generalisable to the wider population we required a sample size of 384 children given 95% confidence and 5% absolute error. We increased the sample size to 500 to obtain ∼55 (unilateral) to a maximum of 110 (bilateral) middle ear discharge specimens for microbiological assessment, on the assumption that 11% of children would have middle ear discharge.^10^

### Statistical analysis

Data were recorded manually on data collection forms, then entered into a REDcap database. The overall study denominator was the total number of children enrolled which included children attending clinic for medical treatment (sick visit), immunisation or ear screening or an accompanying sibling for whom consent was given. In all analyses clinical outcome of ear health assessments was based on the audiologist's final diagnoses for all children with at least one valid ear examination. General data are described using proportions for binary or categorical data and as means (normal) with 95% confidence intervals (CIs) or median (non-parametric) with range for continuous data depending on the data distribution. Child characteristics are presented by reason for attending a clinic. Household density (measure of crowding) was calculated by dividing the number of people by the number of rooms per household. Clinical diagnoses (as recorded by the research nurse) are presented by age group category. OM diagnosis is described overall and by reason for attending clinic (sick visit versus other reasons). The proportion for each diagnostic category (normal, OME, AOM/AOMwiP and CSOM) is shown graphically by reason for attending clinic (sick visit versus other reasons) and by age group. Between-group comparisons were evaluated using logistic regression to estimate odds ratios (OR) in univariable analyses, and adjusted odds ratios (aOR) in multivariable analyses. The multivariable model included all relevant and robust child (including age), maternal, paternal, and household covariates listed in Table 4; only variables considered reliable and accurate, as confirmed by clinical staff, were included. Missing data were addressed using multiple imputation with chained equations (20 imputations), with binary variables imputed using logistic regression, categorical variables with multinomial logistic regression, and continuous variables with linear regression, assuming data were missing at random.^26^ Univariable and multivariable analyses were conducted on the imputed datasets, and estimates were combined using Rubin's rules to produce ORs, aORs and 95% confidence intervals.^27^ All analyses were conducted using Stata software version 17.

### Role of funding source

The funder had no role in study design, collection, analysis, and interpretation of data, writing of the manuscript, and the decision to submit the paper for publication.

## Results

### Description of study participants

Six hundred and forty-six children were triaged at urban outpatient clinics. Seventy-seven percent (498/646) of these were enrolled into the study. Of the 23% (148/646) who were triaged but not enrolled, 87% (129/148) declined their child's participation in the study (many of whom were very unwell and parents were seeking prompt treatment), 10% (15/148) were age-ineligible (>15 years) and the reason for not being enrolled was unknown for 4 children. Of those enrolled, 68% (339/498) attended clinic because they were sick, 15% (76/498) attended for immunisation, 15% (77/498) were siblings of the index child and 6 (<1%) attended for ear screening (Table 1). Children attending for immunisation were the youngest group (median age 0.5 years compared with a median of 5.8 years in siblings of index children and 7.2 years among the 6 attending for ear screening). Eighty-nine percent (68/76) of children attending for immunisation, 19% (64/339) of those attending because they were sick and 10% (8/77) of index children's siblings were aged <12 months (Table 1). Conversely, more sick children (40%, 137/339) and siblings of the index children (60%, 46/77) were aged 5 years or more compared to those attending for immunisation (<1%, 1/339).

**Table 1:** Child characteristics by reason for attending clinic.

	All children (N = 498)	Sick (N = 339)	Immunise (N = 76)	Sibling (N = 77)	Ear screen (N = 6)
	n (%)	n (%)	n (%)	n (%)	n (%)
Female, n (%)	247 (49.6)	170 (50.1)	35 (46.1)	38 (49.4)	4 (66.7)
Age in years, median (range)	3.5 (0.0–15.1)	4 (0.0–15.1)	0.5 (0.0–5.8)	5.8 (0.1–15.1)	7.2 (2.5–9.0)
Age group,					
<6 months	65 (13.1)	21 (6.2)	39 (51.3)	5 (6.5)	0 (0.0)
6–11 months	75 (15.1)	43 (12.7)	29 (38.2)	3 (3.9)	0 (0.0)
1-2 years	91 (18.3)	72 (21.2)	7 (9.2)	11 (14.3)	1 (16.7)
3–4 years	79 (15.9)	66 (19.5)	0 (0.0)	12 (15.6)	1 (16.7)
5–9 years	134 (26.9)	95 (28.0)	1 (1.3)	34 (44.2)	4 (66.7)
≥10 years	54 (10.8)	42 (12.4)	0 (0.0)	12 (15.6)	0 (0.0)
Received ≥2 doses of PCV prior to clinic attendance	189/268 (70.5)	123/161 (76.4)	38/74 (51.4)	26/31 (83.9)	2/2 (100.0)

**PCV:** pneumococcal conjugate vaccine. Children who received PCV but had no date recorded (n = 52) were assigned the median age of receipt based on those with known dates for both doses (n = 208): PCV dose 1, median age 33 days; PCV dose 2, median age 66 days.

The 13vPCV immunisation status was evaluated in 54% (268/498) of children by examining their child health record books. PCV data were missing for almost half the children, generally because parent/guardian had only brought the child's current health record book but not their first book which was full. Available PCV data were evenly distributed across different age groups. Of the children with a valid record, 92% (247/268) had received at least 1 dose of PCV, 78% (208/268) of children had received at least 2 doses at any age, 71% (190/268) before 6 months of age and 71% (189/268) at least 14 days prior to the clinic attendance.

### Presenting complaints

Parents/guardians were asked why they brought their child/children to the clinic. A total of 707 different presenting complaints (average 1.4/child) were reported by parents of 497 enrolled children. Ear pain and/or discharge (24%, 171/707), lower respiratory symptoms (23%, 164/707), fever (15%, 109/707), and abdominal symptoms (10%, 68/707) were the most common. On a per child basis (Supplementary Table S2), 31% (155/497) of parents reported their child had ear pain or ear discharge (or both) while 35% (174/497) of children were reported to have at least one ear symptom or sign (pain, discharge, pulling ears, sores in ear canal, ear wax, foreign body, swelling around the ear, bleeding, injury).

### Clinical diagnoses by research nurse at time of outpatient visit

To provide immediate treatment and/or immunisation to children attending the outpatient clinics, the research nurse made a clinical diagnosis (Supplementary Table S3) following clinical assessment (prior to the independent review of ear health assessments by audiologists in Australia). Three hundred and thirty-nine children presented to the clinic because they were sick, but a clinical diagnosis was recorded on 450 children. This is because mild symptoms (e.g., mild cough, runny nose) were not always volunteered by parent or guardian as part of the clinical history but only noted by the research nurse during the clinical examination when they recorded a clinical diagnosis as appropriate. Multiple illnesses were common (261 children had 1 diagnosis, 156 children had 2 diagnoses, 30 children had 3 diagnoses, 3 children had 4 diagnoses). OM was diagnosed by the research nurses in over half the children at all ages but was most common (77%, 58/75) in 6–11-month-old children. URTIs were most common in the first year of life while lower respiratory tract infections (LRTIs) and gastroenteritis were most common in those aged 6 months to 2 years. OM and/or URTI and/or LRTI were diagnosed in 80% or more of children younger than 3 years, and two-thirds of 3–10-year-olds. When siblings (an older, healthier subgroup) were excluded, age-specific OM patterns remained unchanged, with only small and expected increases in proportion of older children with OM (Supplementary Table S4).

### Proportion of children attending urban clinics with OM

Following the independent ear health assessments by audiologists in Australia, a final middle ear diagnosis was given to 485 children with at least one valid ear examination (Table 2). Approximately three-quarters of these children (74%, 360/485) had some form of OM and 52% (231/444) had bilateral OM. OME was the most common diagnosis (34%, 166/485), 18% (87/485) had AOMwoP while 20% (96/485) had either acute or chronic middle ear discharge (AOMwiP and CSOM). Only 26% (125/485) had normal ears. No cholesteatoma was seen in this study.

**Table 2:** Final otitis media (OM) diagnosis at child level by reason for attendance.

	All children	Sick visit		OR
	(N = 485)	No (N = 154)	Yes (N = 331)	(95% CI)
	n (%)	n (%)	n (%)	
No OM	125 (25.8)	44 (28.6)	81 (24.5)	reference
Any OM	360 (74.2)	110 (71.4)	250 (75.5)	1.23 (0.80–1.90)
OM diagnosis				
OME	166 (34.2)	83 (53.9)	83 (25.1)	0.54 (0.34–0.88)
AOMwoP	87 (17.9)	15 (9.7)	72 (21.8)	2.61 (1.34–5.18)
AOMwiP	41 (8.5)	5 (3.2)	36 (10.9)	3.91 (1.43–10.68)
Dry Perforation	11 (2.3)	1 (0.6)	10 (3.0)	5.43 (0.67–43.84)
CSOM	55 (11.3)	6 (3.9)	49 (14.8)	4.44 (1.76–11.17)
OM broad classification				
Bilateral OM	231/444 (52.0)	72/142 (50.7)	159/302 (52.6)	1.08 (0.73–1.61)
Suppurative OM	183 (37.7)	26 (16.9)	157 (47.4)	4.44 (2.77–7.13)
Middle ear discharge	96 (19.8)	11 (7.1)	85 (25.7)	4.49 (2.32–8.70)
TM perforation	107 (22.1)	12 (7.8)	95 (28.7)	4.76 (2.52–8.99)

Sick visit = Yes: attending for illness. Sick visit = No: attending for other reasons, namely immunisation, ear screening, or sibling of index child. Final OM diagnosis was determined independently by the audiology team in Australia and is based on the child's worst ear. N = 498 had at least one ear exam. N = 485 had a diagnosis recorded. Bilateral OM includes those with a diagnosis in both ears N = 444. **OM:** Otitis media. **OME**: Otitis media with effusion. **AOMwoP**: Acute otitis media without tympanic membrane perforation. **AOMwiP**: Acute otitis media with tympanic membrane perforation. **CSOM**: Chronic suppurative otitis media. **Suppurative OM**: includes AOMwoP, AOMwiP or CSOM. **Middle ear discharge**: includes AOMwiP or CSOM. **Tympanic membrane (TM) perforation:** AOMwiP, Dry perforation or CSOM. Odds ratios (**OR**) were generated using univariate logistic regression. **CI**: Confidence interval. ORs for any OM and the diagnosis categories are relative to findings in children with no OM. ORs for the broader classifications are presented relative to their counterpart (e.g., Suppurative OM versus no Suppurative OM).

### OM diagnosis by age

The severity of OM varied with age (Table 3). OME was most common in children <6 months of age (63%, 39/62), remained high up to 12 months of age (55%, 42/74) and waned thereafter. The more severe forms of OM became prominent and remained so from age 6 months onwards (Table 3). The proportion of children with suppurative OM (AOMwoP, AOMwiP or CSOM, 45%, 39/86), middle ear discharge (29%, 25/86) and TM perforation (34%, 29/86) was highest at 1–2 years of age. Alarmingly, 10% (13/136) of children were diagnosed with CSOM in the first 12 months of life and 19% (16/86) had CSOM at 1–2 years of age.

**Table 3:** Final otitis media (OM) diagnosis by age.

	<6 months (N = 62)	6–11 months (N = 74)	1–2 years (N = 86)	3–4 years (N = 76)	5–9 years (N = 134)	≥10 years (N = 53)
	n (%)	n (%)	n (%)	n (%)	n (%)	n (%)
No OM	13 (21.0)	5 (6.8)	14 (16.3)	24 (31.6)	48 (35.8)	21 (39.6)
Any OM	49 (79.0)	69 (93.2)	72 (83.7)	52 (68.4)	86 (64.2)	32 (60.4)
OM diagnosis						
OME	39 (62.9)	42 (54.8)	29 (33.7)	19 (25.0)	33 (24.6)	4 (7.5)
AOMwoP	4 (6.5)	12 (16.2)	14 (16.3)	17 (22.4)	26 (19.4)	14 (26.4)
AOMwiP	3 (4.8)	5 (6.8)	9 (10.5)	7 (9.2)	11 (8.2)	6 (11.3)
Dry Perf	0 (0.0)	0 (0.0)	4 (4.7)	1 (1.3)	4 (3.0)	2 (3.8)
CSOM	3 (4.8)	10 (13.5)	16 (18.6)	8 (10.5)	12 (9.0)	6 (11.3)
OM broad classifications						
Bilateral OM	34/50 (68.0)	62/69 (89.9)	40/76 (52.6)	35/71 (49.3)	45/126 (35.7)	15/52 (28.8)
Suppurative OM	10 (16.1)	27 (36.5)	39 (45.4)	32 (42.1)	49 (36.6)	26 (49.1)
Middle ear discharge	6 (9.7)	15 (20.3)	25 (29.1)	15 (19.7)	23 (17.2)	12 (22.6)
TM perforation	6 (9.7)	15 (20.3)	29 (33.7)	16 (21.1)	27 (20.1)	14 (26.4)

Final OM diagnosis was determined independently by the audiology team in Australia and is based on the child's worst ear. N = 498 had at least one ear exam. N = 485 had a diagnosis recorded. Bilateral OM includes those with a diagnosis in both ears N = 444. **OM:** Otitis media. **OME**: Otitis media with effusion. **AOMwoP**: Acute otitis media without tympanic membrane perforation. **AOMwiP**: Acute otitis media with tympanic membrane perforation. **CSOM**: Chronic suppurative otitis media. **Suppurative OM**: includes AOMwoP, AOMwiP or CSOM. **Middle ear discharge**: includes AOMwiP or CSOM. **Tympanic membrane (TM) perforation:** AOMwiP, Dry perforation or CSOM.

### OM diagnosis in children attending for illness or for other reasons

**Fig. 1:**
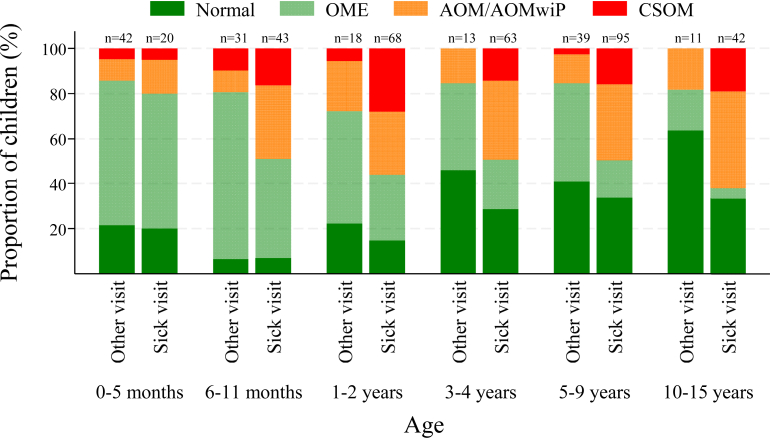
**Ear diagnosis by age among children attending clinic because they were sick** (sick visit) **compared to those attending for other reasons** (Other visit: immunisation, ear check, sibling of index child). OME: otitis media (OM) with effusion; AOM/AOMwiP: acute OM/acute OM with tympanic membrane perforation; CSOM: chronic suppurative OM.

To better understand the burden of ear disease in the study population we evaluated OM among those who presented because they were sick and those who presented for other reasons (siblings or immunisation visits or ear health checks) (Table 2 and Fig. 1). The proportion of children with OM was similar among those presenting with an illness (76%, 250/331) and those presenting for other reasons (71%, 110/154, OR 1.23, 95% CI: 0.80–1.90). Among those who attended for other reasons, OM was present in 58% of siblings (who were generally older) compared to 78% in those attending for immunisation or ear screening. However, OME was less common (OR 0.54, 95% CI: 0.34–0.88) among children who presented because they were sick (25%, 83/331) than among other children (54%, 83/154), indicative of the silent nature of mild disease in generally well children. In contrast suppurative OM (AOM, AOMwiP or CSOM) was more common among those presenting because they were sick (47%, 157/331) than among others (17%, 26/154, OR 4.44 (2.77–7.13)). Specifically, tympanic membrane perforations were more common among those presenting sick than among others (29% 95/331 vs 8% (12/154, OR 4.76 (2.52–8.99))).

The median age of children presenting with an illness (4.0 years, interquartile range (IQR) 1.3–7.2 years) was higher than that of other children (1.2 years, IQR 0.4–6.1 years), largely due to the fact that approximately half (68/140, 49%) of the younger group (age <12 months) presented for immunisation. In view of the differences in reason for attendance between infants (46% < 12months for sickness) and older children (77% ≥ 12 months for sickness), we evaluated OM by age and reason for attendance (Fig. 1). The relative proportion of OME was similar among children presenting because they were sick (60%, 12/20) and those presenting for other reasons (64%, 27/42) children aged <6 months. At older ages, OME accounted for a decreasing proportion of the diagnoses overall but particularly among the children presenting with an illness (Fig. 1, Table 3). In contrast, while the relative prevalence of suppurative OM remained fairly constant with increasing age among the children who presented for reasons other than illness (15%–28%), suppurative OM as a proportion of all OM increased with age among children presenting with an illness (20% at <6 months, 49% at 6–11, 56% at 1–2 years, 49% at 3–4 years, 49% at 3–4 years, 62% at 10–15 years).

### Risk factors for OM

Four hundred and eighty-five children had a valid ear examination. Sixteen percent (81/485) of children had received antibiotics within 30 days of clinic attendance, 36% (167/469) of children's mothers smoked tobacco, 50% (238/477) had completed 10 years of education, 19% (88/475) completed further education and 22% (102/471) were currently employed (Table 4). Compared to mothers, a higher proportion of fathers had completed 10 years of education (59%, 284/479 versus 50%, 238/477, p < 0.01) and further education (39%, 168/433 versus 19%, 88/475, p < 0.001, Table 4). The median number of people per household room was 2.7 (range 0.7–10). Approximately half of families (46%, 219/474) lived in Goroka town. One-third (160/485) of households had a bath or shower, 37% (179/485) used an outdoor tap while 30% (146/485) washed in a river or creek.

**Table 4:** Risk factors for suppurative otitis media (OM).

	All children	Suppurative OM		OR (95% CI)	aOR (95% CI)
	(N = 485)	No (N = 302)	Yes (N = 183)		
	n (%)	n (%)	n (%)		
Child characteristics					
Female	242 (49.9)	141 (46.7)	101 (55.2)	1.41 (0.97–2.03)	1.35 (0.91–2.01)
Age group					
<6 months	62 (12.8)	52 (17.2)	10 (5.5)	reference	reference
6–11 months	74 (15.3)	47 (15.6)	27 (14.8)	2.99 (1.31–6.82)	2.16 (0.86–5.47)
1–2 years	86 (17.7)	47 (15.6)	39 (21.3)	4.31 (1.94–9.59)	4.48 (1.82–11.50)
3–4 years	76 (15.7)	44 (14.6)	32 (17.5)	3.78 (1.67–8.55)	3.71 (1.48–9.29)
5–9 years	134 (27.6)	85 (28.2)	49 (26.8)	3.00 (1.39–6.43)	3.10 (1.32–7.31)
≥10 years	53 (10.9)	27 (8.9)	26 (14.2)	5.00 (2.11–11.89)	4.95 (1.99–12.46)
Received ≥2 doses of PCV prior to clinic attendance	184/259 (71.0)	126/182 (69.2)	58/77 (75.3)	1.04 (0.65–1.66)	0.94 (0.49–1.80)
History of antibiotic use within 30 days of clinic attendance	81 (16.7)	44 (14.6)	37 (20.2)	1.49 (0.92–2.41)	1.78 (1.04–3.06)
Maternal characteristics					
Smokes tobacco	167/469 (35.6)	92/292 (31.5)	75/177 (42.4)	1.56 (1.06–2.31)	1.36 (0.89–2.08)
Completed 10 years of education	238/477 (49.9)	145/297 (48.8)	93/180 (51.7)	1.09 (0.75–1.57)	1.31 (0.80–2.15)
Completed further education	88/475 (18.5)	48/294 (16.3)	40/181 (22.1)	1.39 (0.87–2.21)	1.91 (0.92–3.97)
Currently employed	102/471 (21.7)	61/292 (20.9)	41/179 (22.9)	1.10 (0.71–1.71)	0.80 (0.41–1.53)
Paternal characteristics					
Completed 10 years of education	284/479 (59.3)	185/299 (61.9)	99/180 (55.0)	0.75 (0.51–1.08)	0.93 (0.55–1.57)
Completed further education	168/433 (38.8)	113/270 (41.9)	55/163 (33.7)	0.71 (0.48–1.06)	0.69 (0.40–1.20)
Household characteristics					
People per room, median (range)	2.7 (0.7–10)	2.5 (0.7–10)	3.0 (1–10)	1.12 (1.00–1.25)	1.13 (0.99–1.28)
Location of residence					
Urban (Goroka town)	219/474 (46.2)	144/293 (49.1)	75/181 (41.4)	reference	reference
Peri-urban	132/474 (27.8)	75/293 (25.6)	57/181 (31.5)	1.46 (0.94–2.27)	1.44 (0.86–2.40)
Rural	123/474 (25.9)	74/293 (25.3)	49/181 (27.1)	1.26 (0.80–1.99)	1.25 (0.72–2.15)
Primary washing facilities					
Internal bath or shower	160 (33.0)	102 (33.8)	58 (31.7)	reference	reference
Outside tap	179 (36.9)	115 (38.1)	64 (35.0)	0.98 (0.63–1.53)	0.95 (0.56–1.62)
River or creek	146 (30.1)	85 (28.1)	61 (33.3)	1.26 (0.80–2.00)	1.11 (0.59–2.07)

Table only includes data from children with a final ear diagnosis recorded (N = 485). People per room was assessed in 478 children: 298 with no suppurative OM and 180 with suppurative OM. Where other field data are missing the calculation denominator is included. PCV: pneumococcal conjugate vaccination status was evaluated in 268 children; those who received the vaccine without a date recorded (n = 52) were assigned the median vaccination age of those with known dates: PCV dose 1 (186 known - median age 33 days, 52 unknown); PCV dose 2 (151 known—median age 66 days, 50 unknown). Suppurative OM = No, includes Normal, OM with effusion and dry perforation. Suppurative OM = Yes, includes acute OM with and without perforation and chronic suppurative OM. Odds ratios (**OR**) from univariable and adjusted odds ratios (**aOR**) from multivariable logistic regression are shown, with 95% confidence intervals (CI). Multiple imputation with chained equations (20 imputations) was used to address missing data. ORs are presented relative to the reference group: for binary variables, this is the absence of exposure; for categorical variables, it is the specified reference category; and for continuous variables, ORs represent the change in odds per unit increase in the variable.

Child age and maternal smoking were the only factors significantly associated with any OM diagnosis (versus normal ears, Supplementary Table S5). Compared to children <6 months of age, the risk of any OM was highest at 6–11 months of age (aOR 3.18, 95% CI: 0.95–10.61) and declined steadily thereafter to be lowest among children aged ≥10 years (aOR = 0.35, 95% CI: 0.14–0.86). Children of mothers who smoked tobacco were at higher risk of any OM than children of mothers who did not smoke (aOR 1.80, 95% CI: 1.08–3.00, Supplementary Table S5).

The risk factors for suppurative OM were similar to those for any OM but with a few key differences (Table 4). An age influence was clear but different to that seen with any OM. Compared to children <6 months of age, the risk of suppurative OM was significantly and persistently higher from 1 to 2 years of age onwards ranging from an aOR of 4.48 (95% CI: 1.82–11.50) in children aged 1–2 years to an aOR of 4.95 (95% CI: 1.99–12.46) among those aged ≥10 years. Maternal smoking was not significantly associated with increased risk of suppurative OM in multivariable analysis (aOR 1.36, 95% CI: 0.89–2.08). Children who had received antibiotics in the preceding 30 days were at increased risk of suppurative OM (aOR 1.78, 95% CI: 1.04–3.06). There was a non-significant association between people per household room (metric for crowding) and the risk of suppurative OM (aOR 1.13, 95% CI: 0.99–1.28).

## Discussion

To our knowledge this is the first comprehensive study of ear health in PNG using tympanometry, video-otoscopy and asynchronous telehealth for diagnosis of middle ear disease. We found high rates of OM in all age groups up to age 15 years both among children attending urban clinics because they were sick and among those generally asymptomatic children attending for immunisation or ear screening or who were siblings of the index child. Siblings were included in the study as our study population included any child attending the clinic. Siblings of children with OM may be at increased risk of ear disease and other infections. However, siblings in this study were generally older (Table 1) with lower rates of OM compared to other children (58% vs 78%). OME was the predominant presentation in the first 6 months of life suggesting that early intervention strategies and awareness are essential to prevent progression to the more severe infections seen in older children. In this study, 20% of children had visible middle ear discharge and CSOM affected 1:10 children, with rates mostly >10% from age 6 months onwards, substantially exceeding the World Health Organisation's 4% threshold for declaring an actionable public health emergency.^28^

There has been limited ear health research in the Western Pacific region with which to compare our findings. In our introduction we summarised the findings of Solomon et al.’s study^9^ in urban clinics in Port Moresby, showing lower rates of disease than in our study, due at least in part to only otoscopy being available to detect OM and notably no tympanometer available to detect middle ear effusion. In the Solomon Islands, a study of 288 children aged <3 years attending Child Welfare Clinics in the capital Honiara, Kaspar et al.^29^ reported an overall OM prevalence of 25%, 22% OME, 1.7% CSOM and <1% AOM. Here too the non-availability of tympanometry would in part explain the lower rates compared with our study, particularly for OME (22% vs ∼50% in children younger than 3-years). In the Solomon Islands study OME was more common at age 7–12 months than in younger infants, while in our study OME rates were similar in <6-month and 6–11–months-old children. Tympanometry at 1000 Hz in our study would have increased the likelihood of detecting middle ear fluid from birth to 6 months of age.

The high proportion of children with OM at 1–2 years of age in our study is of particular concern, as it is a time when children should be developing auditory perception in language development, equipping children to be school ready. More research is needed in this area, but we do know that PNG experiences one of the lowest literacy levels in the world, and poor ear health in early life may be one of the contributing factors.^30^

In our study, a high proportion of healthy children were found to have OM, highlighting the need for a better understanding by both families and health workers of the signs and symptoms of OM and where to seek treatment when there are early signs of ear disease. Because OME is frequently asymptomatic, parents are not prompted to seek medical attention. In the PNG context, this is compounded by a weak healthcare system and limited accessibility to health services, often contributing to late presentations. It is therefore essential for health service providers to check ears at every opportunity.^2^^,^^23^ While social determinants play a key role in the early onset of dense bacterial carriage of otopathogens and OM, limited ear and hearing health services due to resource constraints and other competing health priorities likely contribute to the high burden of OM throughout childhood in many Pacific countries.^31^ During the present study, it was reported that health workers could not assess ear health due to a lack of otoscopes in the urban clinics. There were also reports of lack of training of nurses and ENT professionals in the highlands, and their employment was intermittent, further compounding the discontinuity of care for children and their families.

The risk factors identified in this study for any OM were young age and maternal smoking, while increasing age was a risk factor for the more severe suppurative OM (AOM and CSOM) and, while border-line significance, results suggest crowding (people/room) as a potential risk factor. While these are important modifiable risk factors and concur with studies among Aboriginal Australian children,^6^^,^^32^ they are unlikely to fully explain the burden of OM in PNG. The use of open fires for cooking in houses made of bush materials may contribute to the high rates of OM.^33^ Socioeconomic and environmental conditions and poverty have frequently been identified as important contributors to high rates of OM.^15^ An in-depth investigation in the PNG highlands is needed to identify the key contributing factors amenable to prevention. There is an urgent need for targeted interventions that prevent ear disease in this population. Early detection and treatment of OM is needed given the high rates of generally asymptomatic OM in the first 6 months of life.

The study delivered several key methodological strengths. As the first comprehensive study of OM in PNG using advanced diagnostic tools, it established baseline data for policy makers to consider appropriate interventions and for future research. The project built local capacity through training clinical research nurses in tympanometry and video-otoscopy. Additionally, the validation of diagnoses through telehealth consultation with Australian audiologists demonstrated a viable model for remote specialist support. A comparison of the diagnostic accuracy between local nurses and specialist audiologists will be the subject of a future publication. This study also demonstrated the feasibility of otoscopy and tympanometry at 1000 Hz in children during the first 6 months of life, which has historically been considered difficult. The comprehensive training of the Chief Investigator (CA) in Australia with audiologists and ENTs, ongoing support (via Microsoft Teams) and expert review provided by Australian audiologists to clinical staff in PNG enabled an accurate diagnosis to be achieved for most children. Importantly, many children whose illness might not have been detected through routine services benefited from participation in the study and we also hear that there has been enhanced awareness about OM in the community.

The study had several limitations. Our sample consisted of children attending outpatient clinics and therefore may not represent prevalence rates of OM in the wider community. However, we were able to compare the proportion of children with OM among those presenting to clinic because they were sick with the proportion in the generally asymptomatic children attending clinic for other reasons, the latter being somewhat more representative of the general population albeit health service users. We acknowledge that the ‘not-sick’ group is heterogeneous, comprising younger children attending for immunisation and older, generally well siblings. The sensitivity analysis excluding siblings (Supplementary Table S4) demonstrated the expected age effect, with slightly higher proportions of children with OM in some older age groups after removal of this healthier subgroup. However, the age-specific patterns and interpretation of the findings remain unchanged. We also acknowledge the inability to model household clustering because a family identifier was not collected; however, siblings represented a small proportion of the cohort, were generally older and well, and the consistency of results after their exclusion indicates that any residual intra-household correlation is unlikely to have materially influenced our conclusions. A population-based screening study is needed to more accurately estimate the disease burden, but this does not deter from the important findings of this study highlighting unacceptably high rates of OM. Issues of recall and at times non-availability of all parent-held health books, the cross-sectional design and temporal variation in presence of middle ear discharge among those with longstanding perforations made it difficult to differentiate between recurrent AOMwiP and CSOM in this study. We were also unable to evaluate the impact such high rates of OM had on the hearing levels of children. This information would have provided valuable information for parents, clinicians and schools in the community. Audiometry equipment is now available to the PNG team and training of local staff in audiometry has begun. We omitted to ask about or assess balance though no parent/guardian volunteered such a problem. Incomplete PCV data weakened our ability to evaluate its impact on ear disease. In this study, there was a higher representation of urban than peri-urban and rural inhabitants, which may limit the generalisability of the findings to lower resourced rural settings.

Our study marks a significant step forward, but much work has yet to be done. Future research priorities should address several critical knowledge gaps in PNG's ear health. Studies in rural settings are critical to gauge the burden in the lowest socioeconomic regions. A comprehensive school-based study has commenced recently to provide somewhat more representative data on burden of OM in the general paediatric population. Clinical trials are needed to determine optimal CSOM treatment protocols in this setting, including their impact on hearing outcomes and quality of life. Research should also examine the educational and developmental impacts of chronic ear disease in PNG children. Additionally, qualitative research involving families and healthcare providers would provide valuable insights into healthcare-seeking behaviours, cultural perceptions of ear disease, and barriers to care. This mixed-methods approach would inform evidence-based interventions and policy development for both preventive and clinical care strategies. Such research efforts will enable advocacy for ear and hearing health services which are critically lacking in PNG.

In conclusion, this study has found unacceptably high rates of OM among children attending urban outpatient clinics in the Eastern Highlands Province of PNG. In addition to the early onset of ear disease in children aged <6 months, it has shown a high proportion of children have CSOM that requires urgent public health action. The project has built capacity locally through training of clinical and research staff in assessment of ear health and the establishment of a viable model for remote specialist support for ear health, which provided learnings for both PNG and Australian researchers involved in the project.

## Contributors

CA obtained the funding to conduct the study. CA, DL, ML, CBJ, WP, MJB conceived and designed the study. CA, WP, RLF, ML oversaw the implementation of the study. RM led recruitment and data management. RM and PT conducted the clinical examinations. TG and CM provided clinical ear health and paediatric expertise while CBJ and TV provided audiological expertise. TV independently reviewed all audiological data in Australia. MJB led the data analysis conducted together with CA and DL. CA wrote the first draft of the manuscript. All authors had access to all the data in the study and accept responsibility to submit for publication. All authors reviewed the manuscript prior to publication.

## Data sharing statement

The dataset supporting the conclusions of the article is available upon request to the study team.

## Declaration of interests

The study was funded through the Wesfarmers Centre for Vaccines and Infectious Diseases (WCVID) Deborah Lehmann Research Award. Dr Celestine Aho (first author) was the recipient of this award.
