# Supplementary material for: High rates of suppurative otitis media among children attending urban clinics in Goroka, Eastern Highlands Province, Papua New Guinea: a cross-sectional study
Source: Lancet Reg Health West Pac. 2026 Feb 5;67:101807. doi: 10.1016/j.lanwpc.2026.101807 (PMC12906200; doi:10.1016/j.lanwpc.2026.101807)
Supplement: Supplementary Table 1 [file mmc1.docx]

***Supplementary Table* 1: Tympanometry classification used among study participants aged ≥6 months for 226Hz.**

|  | **Ear Canal Volume (ml)** | **Static Compliance (ml)** | **Peak Tympanic Pressure (daPa)** |
| --- | --- | --- | --- |
| Type A | 0.5-1.5 | 0.2-1.6 | +100 to -150 |
| Type B* | 0.5-1.5 | <0.2 | N/A |
| Type B (high)^+^ | >1.5 | N/A | N/A |
| Type C | 0.5-1.5 | 0.2-1.6 | ≤-150 |

^*^Normal ear canal volume, ^+^High ear canal volume. N/A = Not applicable. Note: For children <6 months of age 1000 Hz tympanograms were used, presence of a peak being indicative of normal middle ear function and classified as type A or no peak indicative of OM, classified as type B.^22^
