# Supplementary material for: High rates of suppurative otitis media among children attending urban clinics in Goroka, Eastern Highlands Province, Papua New Guinea: a cross-sectional study
Source: Lancet Reg Health West Pac. 2026 Feb 5;67:101807. doi: 10.1016/j.lanwpc.2026.101807 (PMC12906200; doi:10.1016/j.lanwpc.2026.101807)
Supplement: Supplementary Table 2 [file mmc2.docx]

***Supplementary Table* 2: Presenting complaint as indicated by the child’s parent/guardian.**

|  | **Children**  **(N=497)** |
| --- | --- |
| Upper respiratory symptoms | 30 (6.0) |
| Lower respiratory symptoms | 164 (33.0) |
| Skin problem | 39 (7.8) |
| Ear pain | 83 (16.7) |
| Ear discharge | 83 (16.7) |
| Ear – other* | 26 (5.2) |
| Hearing loss/Developmental delay | 26 (5.2) |
| Fever | 109 (21.0) |
| Abdominal symptoms | 68 (13.7) |
| Urinary symptoms | 1 (0.2) |
| Eye problem | 13 (2.6) |
| Accident/injury | 9 (1.8) |
| Other | 26 (5.2) |

Up to three presenting reason/complaints were recorded per child. One child had missing data. *Reported (n): Pulling ear (2); Swelling (2); Inflamed/sore ear canal (5); ‘Growth ear in canal (2)’; Swelling or growth around ear/mastoid (4); Bleeding/injury (5); Foreign body (4); Smell (1); History of otitis media (1)
