# Supplementary material for: High rates of suppurative otitis media among children attending urban clinics in Goroka, Eastern Highlands Province, Papua New Guinea: a cross-sectional study
Source: Lancet Reg Health West Pac. 2026 Feb 5;67:101807. doi: 10.1016/j.lanwpc.2026.101807 (PMC12906200; doi:10.1016/j.lanwpc.2026.101807)
Supplement: Supplementary Table 3 [file mmc3.docx]

***Supplementary Table* 3: Clinical diagnosis of children recorded by the research nurse in Goroka town by age.**

|  | **All children** |  |  | **Age** |  |  |  |
| --- | --- | --- | --- | --- | --- | --- | --- |
|  |  | **<6 mo** | **6-11 mo** | **1-2 yrs** | **3-4 yrs** | **5-9 yrs** | **≥10 yrs** |
|  | **(N=498)** | **(N=65)** | **(N=75)** | **(N=91)** | **(N=79)** | **(N=134)** | **(N=54)** |
| **Clinic diagnosis** | **n (%)** | **n (%)** | **n (%)** | **n (%)** | **n (%)** | **n (%)** | **n (%)** |
| Otitis Media | 295 (59.2) | 37 (56.9) | 58 (77.3) | 51 (61.5) | 43 (54.4) | 73 (54.5) | 28 (51.8) |
| Hearing loss | 8 (1.6) | 0 (0.0) | 0 (0.0) | 0 (0.0) | 1 (1.3) | 5 (3.7) | 2 (3.7) |
| Other ear conditions | 24 (4.8) | 0 (0.0) | 0 (0.0) | 4 (4.4) | 8 (10.1) | 10 (7.5) | 2 (3.7) |
| URTI | 115 (23.1) | 27 (41.5) | 28 (37.3) | 19 (20.9) | 14 (17.7) | 22 (16.4) | 5 (9.3) |
| LRTI | 61 (12.3) | 6 (9.2) | 17 (22.7) | 21 (23.1) | 7 (7.9) | 9 (6.7) | 1 (1.9) |
| Tuberculosis | 2 (0.4) | 0 (0.0) | 0 (0.0) | 0 (0.0) | 1 (1.3) | 1 (0.8) | 0 (0.0) |
| Scabies | 42 (8.4) | 5 (7.7) | 4 (5.3) | 13 (14.3) | 7 (8.9) | 8 (6.0) | 5 (9.3) |
| Other skin conditions | 17 (3.4) | 0 (0.0) | 2 (2.7) | 3 (3.3) | 7 (8.9) | 2 (1.5) | 3 (5.6) |
| Gastroenteritis | 64 (12.9) | 3 (4.6) | 13 (17.3) | 23 (25.3) | 8 (10.1) | 12 (9.0) | 5 (9.3) |
| Other abdominal conditions | 8 (1.62) | 0 (0.0) | 0 (0.0) | 0 (0.0) | 4 (5.1) | 4 (3.0) | 0 (0.0) |
| Eye problems | 10 (2.0) | 2 (3.1) | 3 (4.0) | 2 (2.2) | 0 (0.0) | 1 (0.8) | 2 (3.7) |
| Injury | 7 (1.4) | 0 (0.0) | 0 (0.0) | 1 (1.1) | 3 (3.8) | 2 (1.5) | 1 (1.9) |
| Other* | 22 (4.4) | 0 (0.0) | 3 (4.0) | 4 (4.4) | 5 (6.3) | 7 (5.2) | 3 (5.6) |
| OM and/or URTI and/or LRTI | 368 (73.9) | 52 (80.0) | 70 (93.3) | 75 (82.4) | 51 (64.6) | 89 (66.4) | 31 (57.4) |
| Healthy – immunization only | 15 (3.0) | 10 (15.4) | 3 (4.0) | 2 (2.2) | 0 (0.0) | 0 (0.0) | 0 (0.0) |
| Healthy – sibling | 29 (5.8) | 1 (1.5) | 1 (1.3) | 2 (2.2) | 6 (7.6) | 15 (11.2) | 4 (7.4) |
| Healthy – ear screen only | 4 (10.8 | 0 (0.0) | 0 (0.0) | 0 (0.0) | 1 (1.3) | 3 (2.2) | 0 (0.0) |

Up to 4 clinical diagnoses were recorded per child per presentation. **URTI**: upper respiratory tract infection. **LRTI**: lower respiratory tract infection. **OM**: otitis media. *Other diagnoses are listed in Supplementary table 5.
