# Supplementary material for: High rates of suppurative otitis media among children attending urban clinics in Goroka, Eastern Highlands Province, Papua New Guinea: a cross-sectional study
Source: Lancet Reg Health West Pac. 2026 Feb 5;67:101807. doi: 10.1016/j.lanwpc.2026.101807 (PMC12906200; doi:10.1016/j.lanwpc.2026.101807)
Supplement: Supplementary Table 4 [file mmc4.docx]

***Supplementary Table* 4: Final otitis media (OM) diagnosis by age (excluding siblings).**

|  | **<6 months**  **(N=58)** | **6-11 months**  **(N=71)** | **1-2 years**  **(N=76)** | **3-4 years**  **(N=64)** | **5-9 years**  **(N=100)** | **≥10 years**  **(N=42)** |
| --- | --- | --- | --- | --- | --- | --- |
|  | **n (%)** | **n (%)** | **n (%)** | **n (%)** | **n (%)** | **n (%)** |
| No OM | 12 (20.7) | 5 (7.0) | 10 (13.2) | 19 (29.7) | 33 (33.0) | 14 (33.3) |
| Any OM | 46 (79.3) | 66 (93.0) | 66 (86.8) | 45 (70.3) | 67 (67.0) | 28 (66.7) |
| ***OM diagnosis*** |  |  |  |  |  |  |
| *OME* | 37 (63.8) | 40 (56.3) | 27 (35.5) | 14 (21.9) | 19 (19.0) | 2 (4.8) |
| *AOMwoP* | 4 (6.9) | 11 (15.5) | 11 (14.5) | 15 (23.4) | 22 (22.0) | 13 (31.0) |
| *AOMwiP* | 3 (5.2) | 5 (7.0) | 9 (11.8) | 7 (10.9) | 10 (10.0) | 5 (11.9) |
| *Dry Perf* | 0 (0.0) | 0 (0.0) | 4 (5.3) | 1 (1.6) | 4 (4.0) | 2 (4.8) |
| *CSOM* | 2 (3.5) | 10 (14.1) | 15 (19.7) | 8 (12.5) | 12 (12.0) | 6 (14.3) |
| ***OM broad classifications*** |  |  |  |  |  |  |
| Bilateral OM | 32/47 (68.1) | 60/66 (90.9) | 35/67 (52.2) | 29/59 (49.2) | 37/93 (39.8) | 15/41 (36.6) |
| Suppurative OM | 19 (15.5) | 26 (36.6) | 35 (46.1) | 30 (46.9) | 44 (44.0) | 24 (57.4) |
| Middle ear discharge | 5 (8.6) | 15 (21.1) | 24 (31.6) | 15 (23.4) | 22 (22.0) | 11 (26.2) |
| TM perforation | 5 (8.6) | 15 (21.1) | 28 (36.8) | 16 (25.0) | 26 (26.0) | 13 (31.0) |

Final OM diagnosis was determined independently by the audiology team in Australia and is based on the child’s worst ear. N=411 had a diagnosis recorded. Bilateral OM includes those with a diagnosis in both ears N=373. **OM:** Otitis media. **OME**: Otitis media with effusion. **AOMwoP**: Acute otitis media without tympanic membrane perforation. **AOMwiP**: Acute otitis media with tympanic membrane perforation. **CSOM**: Chronic suppurative otitis media. **Suppurative OM**: includes AOMwoP, AOMwiP or CSOM. **Middle ear discharge**: includes AOMwiP or CSOM. **Tympanic membrane (TM) perforation:** AOMwiP, Dry perforation or CSOM.
