# Supplementary material for: High rates of suppurative otitis media among children attending urban clinics in Goroka, Eastern Highlands Province, Papua New Guinea: a cross-sectional study
Source: Lancet Reg Health West Pac. 2026 Feb 5;67:101807. doi: 10.1016/j.lanwpc.2026.101807 (PMC12906200; doi:10.1016/j.lanwpc.2026.101807)
Supplement: Supplementary Table 5 [file mmc5.docx]

***Supplementary Table* 5: Risk factors for any form of otitis media (OM).**

|  | **All children** | **Any OM** | |  |  |
| --- | --- | --- | --- | --- | --- |
|  | **(N=485)** | **No**  **(N=125)** | **Yes**  **(N=360)** | **OR**  **(95% CI)** | **aOR**  **(95% CI)** |
|  | **n (%)** | **n (%)** | **n (%)** |  |  |
| **Child characteristics** |  |  |  |  |  |
| Female | 242 (50) | 57 (46) | 185 (51) | 1.26 (0.84-1.90) | 1.27 (0.81-2.00) |
| Age group |  |  |  |  |  |
| *<6 months* | 62 (13) | 13 (10) | 49 (14) | reference | reference |
| *6-11 months* | 74 (15) | 5 (4) | 69 (19) | 3.66 (1.23-10.94) | 4.02 (1.22-13.24) |
| *1-2 years* | 86 (18) | 14 (11) | 72 (20) | 1.36 (0.59-3.15) | 2.06 (0.77-5.53) |
| *3-4 years* | 76 (16) | 24 (19) | 52 (14) | 0.58 (0.26-1.25) | 0.74 (0.30-1.86) |
| *5-9 years* | 134 (28) | 48 (38) | 86 (24) | 0.48 (0.23-0.96) | 0.60 (0.25-2.40) |
| *≥10 years* | 53 (11) | 21 (17) | 32 (9) | 0.40 (0.18-0.92) | 0.39 (0.14-1.05) |
| Received ≥2 doses of PCV prior to clinic attendance. | 184/259 (71) | 49/64 (77) | 135/195 (69) | 0.69 (0.36-1.32) | 0.54 (0.24-1.17) |
| **Maternal characteristics** |  |  |  |  |  |
| Smokes tobacco | 167/469 (36) | 28/121 (23) | 139/348 (40) | 2.21 (1.38-3.55) | 1.95 (1.16-3.27) |
| Completed 10 years of education | 238/477 (50) | 55/123 (45) | 183/354 (52) | 1.32 (0.88-2.00) | 1.14 (0.64-2.04) |
| Completed further education | 88/475 (19) | 23/121 (19) | 65/354 (18) | 0.96 (0.57-1.62) | 0.94 (0.40-2.21) |
| Currently employed | 102/471 (22) | 28/123 (23) | 74/348 (21) | 0.92 (0.56-1.50) | 0.98 (0.45-2.10) |
| **Paternal characteristics** |  |  |  |  |  |
| Completed year 10 | 284/479 (59) | 72/124 (58) | 212/355 (60) | 1.07 (0.71-1.62) | 1.06 (0.55-2.07) |
| Completed further education | 168/433 (39) | 51/112 (46) | 117/321 (36) | 0.69 (0.44-1.06) | 0.63 (0.34-1.19) |
| **Household characteristics** |  |  |  |  |  |
| People per room, median (range) | 3 (1,10) | 3 (1-9) | 3 (1-10) | 1.11 (0.97-1.27) | 1.03 (0.89-1.20) |
| Location of residence |  |  |  |  |  |
| *Urban (Goroka town)* | 219/474 (46) | 59/121 (49) | 160/353 (45) | reference | reference |
| *Peri-urban* | 132/474 (28) | 29/121 (24) | 103/353 (26) | 1.31 (0.79,2.18) | 1.19 (0.65-2.19) |
| *Rural* | 123/474 (26) | 33/121 (27) | 90/353 (26) | 1.01 (0.61,1.66) | 1.15 (0.62-2.15) |
| Primary washing facilities |  |  |  |  |  |
| *Internal bath or shower* | 160 (33) | 45 (36) | 115 (32) | reference | reference |
| *Outside tap* | 179 (37) | 38 (30) | 141 (39) | 1.45 (0.88-2.38) | 1.15 (0.63-2.12) |
| *River or creek* | 146 (30) | 42 (34) | 104 (29) | 0.96 (0.59-1.59) | 0.73 (0.36-1.48) |

Table only includes data from children with a final ear diagnosis recorded (N=485). People per room was assessed in 478 children: 128 with no OM and 354 with OM. Where other field data are missing the calculation denominator is included. **PCV**: pneumococcal conjugate vaccine status was evaluated in 268 children; those who received the vaccine without a date recorded (n=52) were assigned the median vaccination age of those with known dates: PCV dose 1 (186 known - median age 33 days, 52 unknown); PCV dose 2 (151 known – median age 66 days, 50 unknown). **Any OM**: any OM diagnosis. Odds ratios were generated using univariate (**OR**) and multivariate (**aOR**) logistic regression. **CI**: Confidence interval. Unknown or missing fields were coded and kept in regression analysis. The multivariate regression denominator was 478. ORs for categorical variables are presented relative to the reference group.
