# Supplementary material for: High rates of suppurative otitis media among children attending urban clinics in Goroka, Eastern Highlands Province, Papua New Guinea: a cross-sectional study
Source: Lancet Reg Health West Pac. 2026 Feb 5;67:101807. doi: 10.1016/j.lanwpc.2026.101807 (PMC12906200; doi:10.1016/j.lanwpc.2026.101807)
Supplement: Supplementary Table 6 [file mmc6.docx]

***Supplementary Table* 6: Classification of diseases according to International Classification of Diseases 10 codes.^21^**

| **Condition** | **ICD 10 coding** |
| --- | --- |
| Upper respiratory tract infection | J00, J06.9, R05 |
| Lower respiratory tract infection | J18.9, J20.9, J22 |
| Tuberculosis | A16, A18.2 |
| Scabies | B86 |
| Other skin problems | B09, L02.1, L02.3, L02.8, L02.9, L08.8, L24.9, L92.9, R21 |
| Otitis media | H65.9, H66.0, H66.3, H70.0, H70.9, H72, H72.9 |
| Hearing loss | H90.2, H91.9 |
| Other ear problems | H60, H60.3, H60.9, H61.2, H69.9, H73.9, H92, H93.9, T16 |
| Ear screen | Z13.5 |
| Gastroenteric infections | A00-A09.9, A01, A01.0, A09, R10 |
| Other abdominal issues, including worms | B76.9, B82, R10 |
| Eye problems | H01.9, H10.9, H17.9 |
| Injury | S05.9, S31.8, S63.0, S83.0, S93.0, T11.9, T22.2 |
| Immunization | Z27.9 |
| Healthy sibling | Z76.3 |
| Other, includes:  Hepatitis B, oral thrush, malaria, malnutrition, tooth cyst, tooth abscess, umbilical hernia, urinary tract infection, down syndrome, epistaxis, swollen lymph node | B16, B37.0, B37, B50.9, E46, K02.9, K04.7, K42, N39.0, Q90.9, R04.0, R59.0 |
